# Appendicitis

**Published:** 1900-06-16

**Authors:** 


### APPENDICITIS.

DR. JOSEPH WIENER, jun., of New York, has endeavoured to formulate a scheme to assist in the determination of the question, When shall we operate for appendicitis? He fully recognises the uselessness of hard and fast rules, and holds strongly that each case must be decided on its own merits; but he thinks that the following grouping will be found useful, and that, although it must not be supposed that every case will come under one or other of these headings, the great majority of them will do so. He very truly says that, although the time may come when we may be able to diagnose the exact pathological process going on in the appendix before operating, such a feat is out of the question at the present time, and that all our efforts must be centred on the question, Shall we operate? and in regard to this he would summarise the position as follows:—

I. Not every case of appendicitis should be operated on.

II. After a first mild attack try regulation of diet and salines.

III. After a first severe attack remove the appendix.

IV. After two or more even mild attacks operate.

V. In an acute attack (1) do not give opium or morphine. (2) Operate during an attack; (*a*) if a chill manifests itself; (*b*) if the pain is severe enough to require morphine; (*c*) if the pulse is very small; (*d*) if there is persistent vomiting; (*e*) if there is persistent rigidity of the abdominal walls; (*f*) if an abscess can be felt; (*g*) if the general condition makes it imperative; (*h*) if in doubt.

